# Self-reported impact of the COVID-19 pandemic and lockdown on young patients with tic disorders: findings from a case–control study

**DOI:** 10.1007/s10072-022-05997-x

**Published:** 2022-03-11

**Authors:** Cristiano Termine, Vera Galli, Linda Greta Dui, Valentina Berlusconi, Rachele Taras, Marta Vergani, Francesca Lunardini, Simona Ferrante, Andrea Eugenio Cavanna

**Affiliations:** 1 Child Neuropsychiatry Unit, Department of Medicine and Surgery, University of Insubria, Varese, Italy; 2 Department of Maternal and Child Health, Del Ponte Hospital, Varese, Italy; 3 Child Neuropsychiatry Unit, Department of Clinical and Experimental Sciences, University of Brescia, Brescia, Italy; 4 Department of Electronics, Information and Bioengineering, NearLab, Politecnico Di Milano, Milan, Italy; 5 Paediatric Unit, Department of Medicine and Surgery, University of Pavia, Pavia, Italy; 6 Department of Neuropsychiatry, The Barberry National Centre for Mental Health, BSMHFT and University of Birmingham, 25 Vincent Drive, Birmingham, B152FG UK; 7 School of Life and Health Sciences, Aston University, Birmingham, UK; 8 University College London and Institute of Neurology, London, UK

**Keywords:** Adolescents, Children, COVID-19 pandemic, Lockdown, Tic disorders, Tics

## Abstract

**Background:**

Little is known about the perceived impact of the COVID-19 pandemic and subsequent lockdown measures on young patients with tic disorders. Previous studies focused on clinician and parent ratings of tic severity, whereas the only international self-report data are available for adult populations. We present the first findings from a case–control study on children and adolescents with tics during lockdown in Italy.

**Methods:**

We surveyed 49 patients aged 6–18 years and 245 matched controls with a newly developed questionnaire covering socio-demographic and clinical data, as well as lockdown-related changes to daily life activities.

**Results:**

About half (53.2%) of the Italian school-age patients who took part in our survey experienced changes in tic severity during lockdown. Perceived increases in tic severity (29.8%) were reported more often than decreases (23.4%). Analogous trends were reported for perceived restlessness and, more significantly, irritability, whereas changes in pain symptoms were less common and were similar in both directions. The presence of tics was associated with increased difficulties with remote learning (*p* = 0.01), but decreased feelings of missing out on social interactions with schoolmates (*p* = 0.03).

**Conclusions:**

Self-reported data on the impact of COVID-19 lockdown in school-age patients with tic disorders indicate perceived changes in tic severity, as well as restlessness and irritability, in about half of the cases. These findings could guide both clinicians and teachers in the implementation of targeted adjustments in the delivery of care and educational strategies, respectively.

## Introduction

Tic disorders are the most common hyperkinetic movement disorders in childhood [1]. Tics are defined as movements (motor tics) or sounds (phonic tics) that are brief and rapid and occur intermittently and involuntarily. Tics are characteristically preceded by premonitory urges and are known to be modulated by both environmental and psychological factors [2]. It has been documented that underlying anxiety and stress are common tic-exacerbating factors [1]. At the beginning of 2020, the rapid spread of COVID-19 from China to the rest of the world resulted in a global pandemic. Lockdown measures were implemented by most countries in order to contain viral circulation and transmission. In Italy, nationwide school closedown was ordered on March 5th, 2020. As a result, students and teachers were required to switch to distance-learning programmes. The impact of the pandemic and subsequent lockdown on young patients with tic disorders are largely unknown [3, 4]. Two studies conducted in Italy during the COVID-19 pandemic assessed clinician-rated tic severity [5] and parent-reported tic severity [6], respectively, reporting somewhat conflicting findings. We present the first self-reported data from a case–control study on young patients with tic disorders.

## Methods

During the first COVID-19 Italian lockdown period (April–June 2020), we developed an online questionnaire (85 questions) covering socio-demographic and clinical data, as well as lockdown-related changes to daily life activities (Table 1).

**Table 1:** Key items from the online questionnaire covering socio-demographic and clinical data, as well as lockdown-related changes to daily life activities and well-being

Socio-demographic and clinical variables	
Socio-economic status	What’s the parents’ employment? (higher executive/business manager/administrative personnel/clerical and sales/skilled manual/semi-skilled/unskilled/never employed)
Parents’ job status	Do parents work as health professionals? (yes, both/yes, one/no)
Contact with infected people	Have you been in touch with COVID-19 positive subjects? (yes/no)
Access to outdoor spaces at home	As part of your own house, do you have access to a terrace/garden? (yes/no)
Tic status: severity	Have you noticed any change in tic severity? (yes, they have improved/yes, they have worsened/no)
Tic status: type	Have you noticed any change in tic type? (yes/no)
Tic-related pain	Have you noticed any change in tic-related pain? (yes, it has improved/yes, it has worsened/no change/never experienced)
Restlessness	Have you noticed any change in restlessness? (yes, it has improved/yes, it has worsened/no change/never experienced)
Irritability	Have you noticed any change in irritability? (yes, it has improved/yes, it has worsened/no change/never experienced)
Daily life activities and well-being	
Stress/anxiety	What is your level of stress/anxiety? (more than before/same as before/less than before)
Changes in daily routines	Have you been able to keep your daily routines? (yes, completely/yes, partially/no)
Changes in social contacts	Have you been able to keep in touch with your friends through social media? (more than before/same as before/less than before)
Time spent using electronic devices	How many hours per day have you been spending using the telephone/tablet/personal computer/television/playstation? (< 2 h/2–6 h/ > 6 h)
Difficulties with remote learning	Have you complied with remotely delivered lectures and assignments? (yes, without difficulty/yes, with difficulty/no)
Interest in school reopening	How often have you asked questions about school reopening? (often/sometimes/never)

The questionnaire was sent to a clinical sample of children and adolescents (age range 6–18 years) with a DSM-5-validated diagnosis of tic disorder (*n* = 49; 39 males). After removal of the clinical items, a shorter version of the questionnaire was sent to a large group of matched healthy controls (*n* = 245; 195 males). The clinical sample was recruited through the local Child and Adolescent Psychiatry Unit (Filippo del Ponte Hospital, Varese, Italy) and the Italian Tourette Syndrome Association, whereas the controls were recruited through the Education Office of the province of Varese, Italy. The study was approved by the local Ethics Committee and all participants gave informed consent before filling their questionnaire anonymously.

Firstly, we focused on self-reported data from the clinical sample to assess changes in the perception of tic severity (as well as restlessness, irritability, and pain) after the lockdown measures were implemented. Secondly, we analysed the controlled data and performed a logistic regression analysis adjusted for age and gender (RStudio, 1.3.1056) to assess the possible impact of socio-demographic and clinical variables (gender, education level, socio-economic status, parents’ job status, family structure, contact with infected people, access to outdoor spaces at home, tic status) on a number of lifestyle and well-being parameters, including stress/anxiety, changes in daily routines and social contacts, time spent using electronic devices, difficulties with remote learning, and interest in school reopening.

## Results

**Fig. 1:**
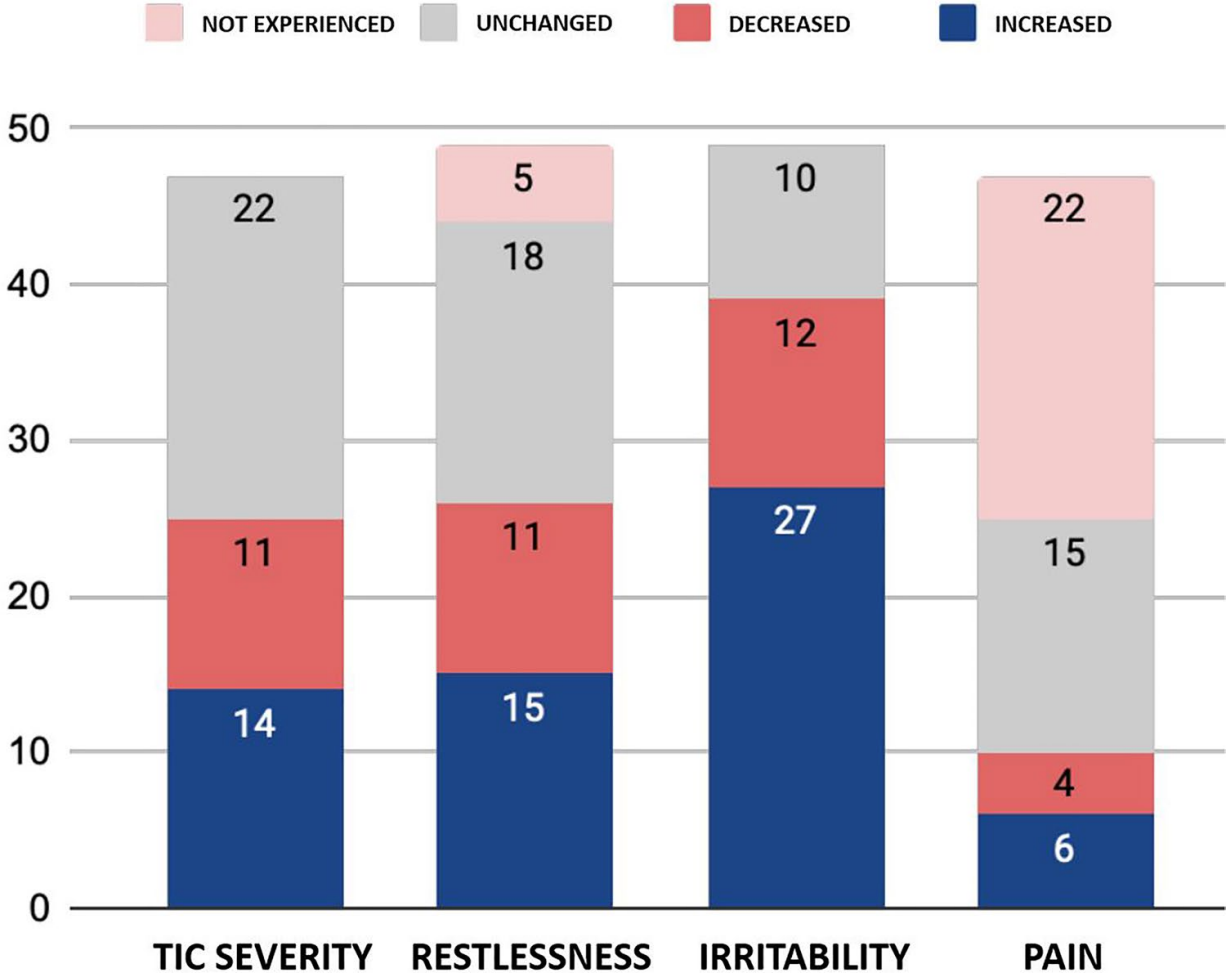
Self-reported changes in tic severity and related clinical variables in young patients with tic disorders during lockdown

About half (53.2%) of the young patients with tic disorders reported changes in tic severity during the lockdown, with 20.4% also reporting changes in tic type. Specifically, 29.8% of the surveyed patients reported tic worsening, whereas 23.4% reported tic improvement (Fig. 1). None of the tested variables was found to be significantly associated with either tic worsening or tic improvement. A similar pattern was observed for perceived changes in overall restlessness (53.1%): increased restlessness was reported by 30.6% of patients, whereas 22.4% reported decreased restlessness. The majority of patients (79.6%) reported changes in irritability (increase in 55.1% of patients and decrease in 24.5%). Changes in pain symptoms were reported by 21.3% of patients: 12.8% perceived increased pain, and 8.5% decreased pain.

According to the regression analysis, the presence of tics was associated with increased difficulties with remote learning (*t* =  − 2.58; *p* = 0.01), but decreased feelings of missing out on social interactions with schoolmates (*t* =  − 2.19; *p* = 0.03). Having access to an outdoor space at home was found to be significantly associated with reduced stress/anxiety and preserved daily routines (*t* = 3.89, *p* < 0.01; and *t* = 2.55, *p* = 0.01, respectively). Participants with parents working as health professionals were more likely to report less stress/anxiety (*t* = 3.19; *p* < 0.01), despite changes in their daily routines (*t* =  − 5.10; *p* < 0.01). Lower socio-economic status was associated with increased stress/anxiety (*t* = 6.14; *p* < 0.01) and interest in school reopening (*t* =  − 2.45; *p* = 0.01). Participants with access to an outdoor space at home were also less likely to report social isolation (*t* = 11.15; *p* < 0.01). Finally, all participants reported increased time spent using electronic devices, regardless of access to outdoor spaces (*t* = 5.78; *p* < 0.01).

## Discussion

According to the first self-reported data from Italian school-age patients with tic disorders, about half of the patients experienced changes in tic severity during lockdown, and about one in five also reported changes in tic type. Perceived increases in tic severity were reported more often than decreases. Similar trends were reported for perceived restlessness and, more significantly, irritability, whereas changes in pain symptoms were less common and took both directions. Two previous studies conducted in Italy assessed the impact of the COVID-19 pandemic on tic severity in children and adolescents. The first study reported an overall reduction in clinician-rated tic severity: this was mainly driven by tic-related impairment scores (− 23%), whereas both motor and phonic tic severity subscores were stable or reduced to a lesser extent [5]. The results of the second study, a survey directed at parents of patients with tic disorders, were more in line with our findings: on average, parent-reported tic severity was found to be increased in both motor tics (42%) and phonic tics (36%) [6]. Our results are also consistent with the results of a different study on self-reported tic severity conducted in adults. In a large survey of 178 adults diagnosed with tic disorders and mostly located in Europe (58%) and North America (35%), approximately half of the participants (48%) experienced that their tic severity had increased during the pandemic [7]. Although all the patients enrolled in our study had a clinician-validated diagnosis of tic disorder, it cannot be ruled out that at least some of the 10 patients who reported changes in tic type might have developed functional tics (‘functional overlay’). This would be consistent with the recent reports of rapid-onset functional tic-like behaviours during the COVID-19 pandemic, often in the context of increased exposure to social media [8, 9].

Most of the associations between socio-demographic/clinical variables and lifestyle/well-being parameters that we observed were in line with expectations. Interestingly, young patients with tics reported fewer feelings of missing out on social interactions with their schoolmates, as compared to healthy controls. This might be due to their difficulties in socialisation, which are related to their low self-esteem [10] and perceived social stigma in the school environment [11]. In addition to the known limitations of the subjective reporting of tic severity [12], a few limitations related to the design of our study should be mentioned: since our analysis was based on the online questionnaire, there is a risk of potentially significant recall bias, as well participant bias, because patients’ presentations could not be confirmed by clinicians’ assessments. Despite such limitations, these original findings should be taken into account by both clinicians and teachers who might need to adjust their management strategies and educational approaches to better address patients’ needs during unprecedented times.
